# Recombinant neorudin and its active metabolite hirudin: the fate *in vivo* of a novel anticoagulant drug

**DOI:** 10.3389/fphar.2024.1443475

**Published:** 2024-09-17

**Authors:** Qiang Li, Yubin Liu, Boyuan Ren, Jiayan Jin, Lin Zhang, ChuTse Wu, JiDe Jin

**Affiliations:** ^1^ Beijing Institute of Radiation Medicine, Beijing, China; ^2^ Division of (Bio) Pharmaceutics, Institute of Zhejiang University - Quzhou, Quzhou, Zhejiang, China; ^3^ The Quzhou Affiliated Hospital of Wenzhou Medical University, Quzhou People’s Hospital, Quzhou, Zhejiang, China

**Keywords:** recombinant neorudin, hirudin, arteriovenous bypass thrombosis, thrombus, peripheral blood

## Abstract

Thrombosis, a prevalent condition, can provoke severe health issues like acute coronary syndrome (ACS), deep vein thrombosis (DVT), and pulmonary embolism (PE). The rising incidence of these diseases annually significantly impacts patient wellbeing and poses a substantial burden on healthcare systems. Recombinant neorudin is a developing anticoagulant drug for thrombotic diseases whose phase I clinical trials has been completed. The distribution pattern of it and its active metabolite, hirudin, in thrombi, blood surrounding the thrombus and peripheral blood remains uncertain. This study explored their distribution using a rat arteriovenous bypass thrombosis model, revealing higher neorudin levels in blood surrounding the thrombus and elevated hirudin concentrations in thrombus. Recombinant neorudin significantly increased Thrombin Time (TT) in both plasma surrounding the thrombus and peripheral blood, and reduced the wet weight of the thrombus. The results above demonstrated the anticoagulant and antithrombotic efficacy of recombinant neorudin *in vivo*. Give the distribution pattern of neorudin and hirudin, we hypothesized that neorudin was cleaved at the site of thrombus formation to produce hirudin, leading to the rapid accumulation of hirudin within local thrombi and resulting in a higher concentration inside the thrombus. This insight was crucial for understanding the action mechanisms of anticoagulants in thrombosis management and provided a valuable guidance for therapeutic strategies in treating thrombotic diseases.

## Introduction

Thrombosis, medically referred to as thromboembolism, is a pathological condition caused by an abnormal coagulation of blood components, where platelets and fibrin form dense clots that may block blood vessels. This phenomenon primarily occurs within the venous or arterial system, leading to diseases including acute coronary syndrome (ACS) (Song et al., 2021), deep vein thrombosis (DVT) (Long and Gottlieb, 2023), pulmonary embolism (PE) (Maughan et al., 2021), coronary artery disease (CAD) (Mayer et al., 2024), and stroke. The mechanism of thrombus formation involves Virchow’s triad: alterations in blood flow (hemodynamic changes), endothelial injury, and changes in the composition of blood (hypercoagulability). These factors work together to promote platelet aggregation and the activation of clotting factors, ultimately resulting in the formation of a thrombus. Globally, thrombosis and thrombosis-related diseases are among the leading causes of death and disability. According to the World Health Organization (WHO), cardiovascular diseases cause approximately 17.9 million deaths worldwide each year, many of which are directly related to thrombosis. Timely diagnosis and effective treatment of thrombosis are crucial for preventing severe complications and reducing mortality rates. The main treatment strategy involves anticoagulation therapy, which is crucial for the prevention and management of thrombus formation (Joglar et al., 2024; Yu et al., 2024). This includes the use of anticoagulants like Warfarin and novel oral anticoagulants (NOACs) such as rivaroxaban, apixaban, and dabigatran (Jun et al., 2023; Sciences et al., 2024; Zhi et al., 2019). These NOACs are recommended by the latest guidelines as first-line treatments due to their favorable risk-benefit profiles, ease of use for patients, and reduced need for monitoring compared to traditional anticoagulants like Warfarin (Connolly et al., 2009; Granger et al., 2011; Patel et al., 2011). Warfarin remains a second-line or alternative therapy for patients with specific indications or contraindications to NOACs. Additionally, platelet inhibitors and thrombolytic therapies are used to reduce the risk of thrombus recurrence, restore vascular patency, and protect organ function (Abbruzzese et al., 2023; Tschudi et al., 2009; Witt et al., 2016). However, these medications can cause serious bleeding events, including gastrointestinal bleeding and intracranial hemorrhage, especially when the dosage is not properly managed or when there are interactions with other drugs (Guyatt et al., 2012; Klok et al., 2015; Shah et al., 2023; Shinge et al., 2022).

Hirudin is a small protein composed of 65 or 66 amino acids and is the most potent natural thrombin inhibitor known to date (Stringer and Lindenfeld, 1992). It has a strong anticoagulant effect and is effective in preventing and treating both arterial and venous thrombosis, more so than heparin (Junren et al., 2021; Markwardt, 1994). Initially, hirudin was approved by the European Medical Evaluation Agency (EMEA) for the treatment of heparin-induced thrombocytopenia (HIT) associated with thrombosis, and later by the U.S. Food and Drug Administration (FDA) for thromboprophylaxis following major orthopedic surgeries (Dong et al., 2020; Gajra et al., 2008; Greinacher and Lubenow, 2001; Piña, 1999). However, the use of hirudin is often associated with severe, even life-threatening bleeding, which limits its clinical application (Zhang and Lan, 2018). The narrow therapeutic window between the clinical benefits and the risks of bleeding greatly restricts the use of hirudin in clinical settings (Eikelboom and French, 2002).

Recombinant neorudin (EPR-hirudin, EH) (Dong et al., 2018; Zhang et al., 2008) is a precursor drug of hirudin that was developed by introducing a short peptide (Glutamic-Proline-Arginine, EPR) that can be recognized and cleaved by coagulation factors FXa and/or FXIa, into the N-terminus of Type II hirudin. EH consists of 68 amino acids and is produce through fermentation and secretion by recombinant pichia pastoris GS115 (Liu, 2018). Compared to hirudin, the only structural difference is at the N-terminus, where EH has three additional amino acids (glutamate, proline and arginine, EPR); the rest of the structure remains the same. The toxicity assessment of neorudin in cynomolgus monkeys demonstrated its low toxicity and high safety (Liu et al., 2023), and its Phase I clinical trial a (registered at chinadrugtrials.org.cn, Clinical Trial Registry number: CTR20160444) had also been completed (Liu et al., 2021). The mechanism of action of EH involves the specific recognition and cleavage by activated coagulation factors FXa and FXIa during local thrombus formation and activation of the coagulation system. This cleavage removes the EPR short peptide from the N-terminus of EH, converting EH from an inactive form to an active anticoagulant hirudin, thus providing targeted antithrombotic action. Essentially, the complete EH has no anticoagulant activity; it only releases hirudin with antithrombotic activity when the coagulation system within the body is activated. Combining preclinical animal studies (Liu et al., 2022a; Wang et al., 2013), it had been demonstrated that EH not only effectively inhibited thrombus formation but also reduced the risk of bleeding by enhancing the specificity of hirudin. It was reported that EPR peptide was also used to link Hirudin Variant III and human serum albumin to construct a fusion protein, proving that this fusion protein was an effective method to limit the bleeding side effects of hirudin (Sheffield et al., 2018).

In this study, we found that recombinant neorudin demonstrated a dose-dependent reduction in thrombus formation in a rat arteriovenous bypass thrombosis model. Notably, the concentration of neorudin in peripheral blood plasma, the plasma adjacent to the thrombus, and within the thrombus homogenate escalated in tandem with dosage levels. Intriguingly, neorudin concentrations were notably higher in blood surrounding the thrombus and in peripheral blood compared to those within the thrombus homogenate. Additionally, hirudin can be detected in the peripheral blood plasma, the plasma surrounding the thrombus, and in the thrombus homogenate, with slightly higher levels of hirudin in the plasma surrounding the thrombus and in the thrombus homogenate than in the peripheral blood plasma. Overall, recombinant neorudin manifested its anticoagulant and antithrombotic properties primarily at the thrombus site, thereby minimizing interference with the coagulation system in areas devoid of thrombotic activity and consequently reducing the likelihood of bleeding complications.

## Materials and methods

### Standard samples and standard reference materials

Standard samples: recombinant neorudin for injection (Yeast), standard samples form: lyophilized powder for injection, molecular weight: 7.3 kDa, activity: >10,240 ATU, specification: 20 mg/vial, storage conditions: store at 2°C–8°C, packaging: sealed in a vial made of glass, with a rubber stopper and aluminum foil seal.

Positive control drug: hirudin, standard reference form: lyophilized powder for injection, molecular weight: 7.0 kDa, activity: >10,240 ATU, specification: 10.32 mg/vial, storage conditions: store at 2°C–8°C, packaging: sealed in a vial made of glass, with a rubber stopper and aluminum foil seal.

## Reagents

Isoflurane (Batch number 20220502), prothrombin time (PT) Assay Kit (Batch number 012201A), thrombin time (TT) Assay Kit (Batch number 032103B), and activated partial thromboplastin time (APTT) Assay Kit (Batch number 022207A) were purchased from MD Pacific (Tianjing, China).

## Animals

Male Sprague-Dawley (SD) rats, 9 weeks of age (SCXK (Beijing) 2019-0010), were used in this animal experiment (Liu et al., 2022a; Liu et al., 2023). The study has been reviewed and approved by Institutional Animal Care and Use Committee (IACUC) of Yoji (Tianjin) Medical Laboratory Animal Ethics Committee, with the approval number of IACUC20221022-PD01.00. The experimental procedures were conducted strictly in accordance with the requirements of IACUC to ensure animal welfare.

All animals were housed in standard housing areas with a temperature of 20°C–26°C and humidity of 40%–65%. During the housing process, a maximum of 5 animals were kept in each cage, and they were fed twice a day. Throughout the experiment, all animals involved were provided with free access to water. Each cage containing animals was clearly and specifically labeled.

## Methods

### Model preparation

The rats were placed in a supine position and fixed on the operating table after anesthesia with isoflurane. The skin in the midline of the rat’s neck was incised, and the fascia was separated to expose the left carotid artery and right external jugular vein. A polyethylene tube with an embedded silk thread was used for arterial-venous shunt surgery. The artery clamp was used to clamp the carotid artery near the heart, and one end of the polyethylene tube was inserted into the artery and the other end into the vein to complete the shunt surgery.

## Grouping and drug administration

The experimental animals were systematically allocated into six groups, each consisting of eighteen animals (n = 18), based on their body weights ranging from 280 to 320 g. The groups were designated as follows: the model control group, which received a continuous intravenous infusion of normal saline, and the test drug groups, which were administered continuous intravenous infusions of neorudin at escalating doses of 3 mg/kg/h, 6 mg/kg/h, 12 mg/kg/h, and 18 mg/kg/h, respectively. Additionally, the positive control group was treated with a continuous infusion of hirudin at a dose of 2.88 mg/kg/h, an equimolar dose corresponding to 3 mg/kg of neorudin. For all groups, the infusion volume was maintained at 2 mL/kg, with the infusion period lasting for 1 h.

In the rat arteriovenous shunt model, prior to inducing thrombus formation by opening the artery clamp, a bolus injection of 2.5 mg/kg of neorudin or hirudin was administered intravenously. The artery clamp was then opened to induce thrombus formation. Thrombi, blood around the thrombus, and peripheral plasma from the jugular vein were collected for analysis at 15 min, 1 h, and 1 h after the discontinuation of the drug in each group. The details of the grouping and drug administration protocols are summarized in Table 1.

**TABLE 1:** Group and drug administration for arteriovenous shunt thrombus model.

Group	Dosage	Time of administration	15 min after administration	1 h after administration	1 h after withdrawal
Model	NS	Simultaneous administration of the drug was initiated during thrombotic induction	Per group n = 6	Per group n = 6	Per group n = 6
Neorudin	3 mg/kg/h	Simultaneous administration of the drug was initiated during thrombotic induction	Per group n = 6	Per group n = 6	Per group n = 6
Neorudin	6 mg/kg/h	Simultaneous administration of the drug was initiated during thrombotic induction	Per group n = 6	Per group n = 6	Per group n = 6
Neorudin	12 mg/kg/h	Simultaneous administration of the drug was initiated during thrombotic induction	Per group n = 6	Per group n = 6	Per group n = 6
Neorudin	18 mg/kg/h	Simultaneous administration of the drug was initiated during thrombotic induction	Per group n = 6	Per group n = 6	Per group n = 6
Hirudin	2.88 mg/kg/h	Simultaneous administration of the drug was initiated during thrombotic induction	Per group n = 6	Per group n = 6	Per group n = 6

### The preparation and storage of the test substances are as follows

Recombinant Neorudin Preparation: dissolve the powder in 1 mL of sterile water for injection, creating a solution with a concentration of 19.5 mg/mL.

## Measurement of thrombus weight

At 15 min and 1 h after continuous administration, and 1 h after discontinuation of the drug, the blood flow in the arteriovenous bypass was interrupted, and the thrombus wrapped in cotton thread was quickly removed. After removing residual blood with filter paper, the wet weight of the thrombus was measured. The thrombus was then prepared into a 10% thrombus homogenate using normal saline (NS).

### Coagulation testing of thrombus surrounding blood and peripheral blood

At 15 min and 1 h after continuous administration, and 1 h after discontinuation of the drug, 0.5 mL of thrombus surrounding blood and peripheral blood were collected. The blood samples were anticoagulated with 3.8% sodium citrate, and centrifuged at 4°C and 3,000 rpm for 10 min to obtain plasma. Thrombus surrounding blood plasma was tested for TT, and peripheral blood plasma was tested for APTT, TT and PT.

### Drug distribution testing

50 µL of plasma from blood surrounding the thrombus, peripheral blood plasma, and homogenized thrombus solution was collected separately to detect the concentrations of recombinant neorudin and hirudin using liquid chromatography-tandem mass spectrometry (LC-MS/MS) (Dong et al., 2018; Dong et al., 2017). Detailed parameters were shown in Table 2; Supplementary Figure S1.

**TABLE 2:** The mass spectrometry details.

Parameter	Neorudin	Hirudin	Internal standard
Detection method	ESI+; MRM	ESI+; MRM	ESI+; MRM
Declustering Potential(V)	120.000	95.000	125.000
Collision Energy(V)	41.000	32.000	41.000
Entrance Potential(V)	10.000	10.000	10.000
Collision Cell Exit Potential (V)	20.000	20.000	20.000
Collision Gas (μL/min)	12	12	12
Curtain gas (μL/min)	40.0	40.0	40.0
Ion Source Gas1 (μL/min)	50.0	50.0	50.0
Ion Source Gas2 (μL/min)	70.0	70.0	70.0
IonSpray Voltage (V)	5,500.0	5,500.0	5,500.0
Temperature (°C)	600.0	600.0	600.0
m/z	1,216.000→1,303.600	1,152.100→1,353.300	1,237.900→1,330.100

Note: LC-MS/MS, SHIMADZU LC-20AD- API4000 (Analyst 1.6.2).

### Data processing and statistical analysis

To compare two distinct groups, the unpaired Student’s t-test was employed, while the comparison among multiple groups was facilitated through one-way ANOVA, accompanied by either Dunnett’s *post hoc* test or Sidak’s *post hoc* test, utilizing GraphPad Prism (USA). The data are expressed as the mean ± SEM.

## Results

### Thrombus wet weight

During a 15-min period of continuous administration, neorudin was administered at doses of 3 mg/kg, 6 mg/kg, 12 mg/kg, and 18 mg/kg, resulting in a dose-dependent decrease in thrombus wet weight. The reductions observed were 17.8% (*P* > 0.05), 29.7% (*P* < 0.01), 30.3% (*P* < 0.01), and 35.9% (*P* < 0.01), respectively. Notably, hirudin, at a dose of 2.88 mg/kg, exhibited a significant reduction in thrombus wet weight (*P* < 0.01).

When the administration was extended to 1 h, the dose-dependent effect of neorudin on reducing thrombus wet weight continued, with reductions of 4.10% (*P* > 0.05), 20.1% (*P* > 0.05), 31.9% (*P* < 0.05), and 34.6% (*P* < 0.01) for the respective doses. Hirudin, at a dose of 2.88 mg/kg, significantly reduced thrombus wet weight, with a marked decrease (*P* < 0.01).

**FIGURE 1:**
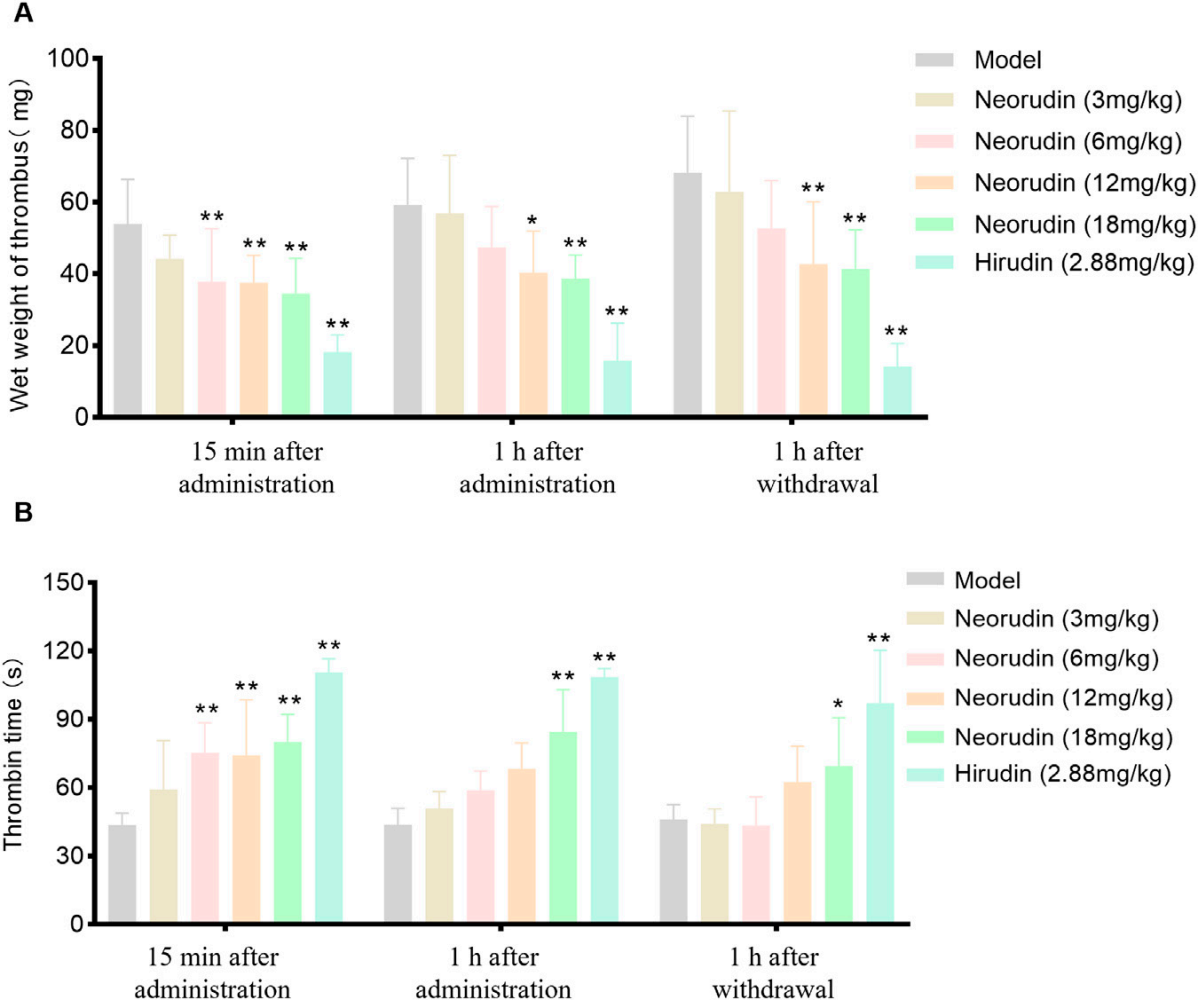
Thrombus wet weight and TT in plasma surrounding the thrombus (n = 6 rats per group). **(A)** Visuals of wet weight of thrombus (mg). **(B)** Visuals of perithrombotic blood TT (s). (Error bars, mean ± SEM from biological replicates. Compared to the model control group: *P < 0.05, **P < 0.01). TT: thrombin time.

One hour after discontinuation, the administration of neorudin in doses of 3 mg/kg, 6 mg/kg, 12 mg/kg, and 18 mg/kg still showed a continued dose-dependent reduction in thrombus wet weight. The decreases recorded were 7.90% (*P* > 0.05), 22.7% (*P* > 0.05), 37.4% (*P* < 0.05), and 39.4% (*P* < 0.01), respectively. In contrast, hirudin, at a dose of 2.88 mg/kg, significantly diminished thrombus wet weight, with a notable reduction (*P* < 0.01) (Figure 1A).

### Measurement of TT at the plasma around thrumbus

#### Detection of TT in perithrombus plasma

During a 15-min continuous administration period, hirudin at a dose of 2.88 mg/kg significantly prolonged TT to 110.7 s (*P* < 0.01), while neorudin, at a dose of 3 mg/kg, did not significantly affect TT (59.2 s). However, higher doses of neorudin at 6 mg/kg, 12 mg/kg, and 18 mg/kg markedly prolonged TT to 75.3 s, 74.2 s, and 80.1 s, respectively (*P* < 0.01).

With the extension of administration to 1 h, hirudin maintained its significant impact on TT at a dose of 2.88 mg/kg, with a value of 108.5 s (*P* < 0.01). However, neorudin at doses of 3 mg/kg, 6 mg/kg, and 12 mg/kg did not significantly alter TT, with values of 51.0 s, 58.8 s, and 68.3 s, respectively, while the 18 mg/kg dose significantly extended TT to 84.4 s (*P* < 0.01).

One hour following discontinuation, the effect of hirudin at 2.88 mg/kg on prolonging TT remained significant, with a value of 97.1 s (*P* < 0.01). At this point, neorudin at doses of 3 mg/kg, 6 mg/kg, and 12 mg/kg did not show a significant impact on TT, with values of 44.2 s, 43.3 s, and 62.5 s, respectively. However, a dose of 18 mg/kg was effective in significantly prolonging TT to 69.6 s (*P* < 0.05) (Figure 1B).

#### Detection of coagulation parameter in peripheral plasma

During a 15-min period of continuous administration, hirudin at a dose of 2.88 mg/kg significantly extended TT to 102.7 s (*P *< 0.01). In contrast, equimolar doses of neorudin at 3 mg/kg, as well as higher doses of 6 mg/kg, 12 mg/kg, and 18 mg/kg, did not significantly affect TT, with values of 45.5 s, 51.2 s, 53.2 s, and 57 s, respectively.

When the administration was prolonged to 1 h, hirudin continued to significantly prolong TT at a dose of 2.88 mg/kg, with values of 105.1 s (*P *< 0.01). At this juncture, the 3 mg/kg dose of neorudin modestly extended TT, with a value of 49.4 s, while the doses of 12 mg/kg and 18 mg/kg significantly lengthened TT, with values of 57.3 s and 60.9 s, respectively (*P *< 0.01).

**FIGURE 2:**
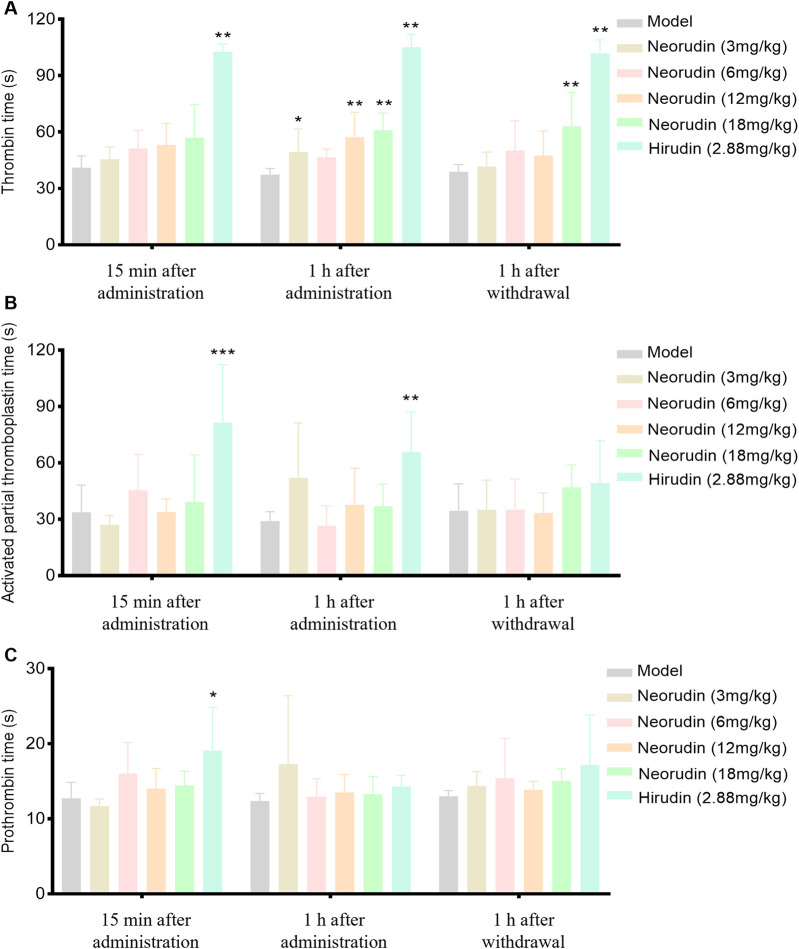
TT, APTT and PT in rat peripheral blood plasma (n = 6 rats per group). **(A)** Visuals of peripheral blood TT (s). **(B)** Visuals of peripheral blood APTT (s). **(C)** Visuals of peripheral blood PT (s). (Error bars, mean ± SEM from biological replicates. Compared to the model control group: *P < 0.05, **P < 0.01, ***P < 0.001). TT: thrombin time; APTT: activated partial thromboplastin time; PT: prothrombin time.

One hour after discontinuation, the impact of hirudin at 2.88 mg/kg on TT remained significantly prolonged, with values of 101.9 s (*p *< 0.01). The doses of 3 mg/kg, 6 mg/kg, and 12 mg/kg of neorudin showed no significant effect on TT, with values of 41.6 s, 50.2 s, and 47.4 s, respectively. However, the 18 mg/kg dose of neorudin significantly extended TT to 60.1 s (*P *< 0.01) (Figure 2A).

Continuous administration of hirudin, both for 15 min and 1 h, demonstrated a significant effect on APTT compared to the model control group, with APTT values increasing to 81.4 s and 65.7 s, respectively (*P *< 0.01), while neorudin had no significant effect on APTT (Figure 2B). At the same time, neorudin administration did not affect significantly the value of PT, contrasting that hirudin showed an evident PT prolongation to 19.1 s (*P *< 0.01) at 15 min after treatment (Figure 2C).

#### Detection of neorudin and hirudin in peripheral blood plasma

**FIGURE 3:**
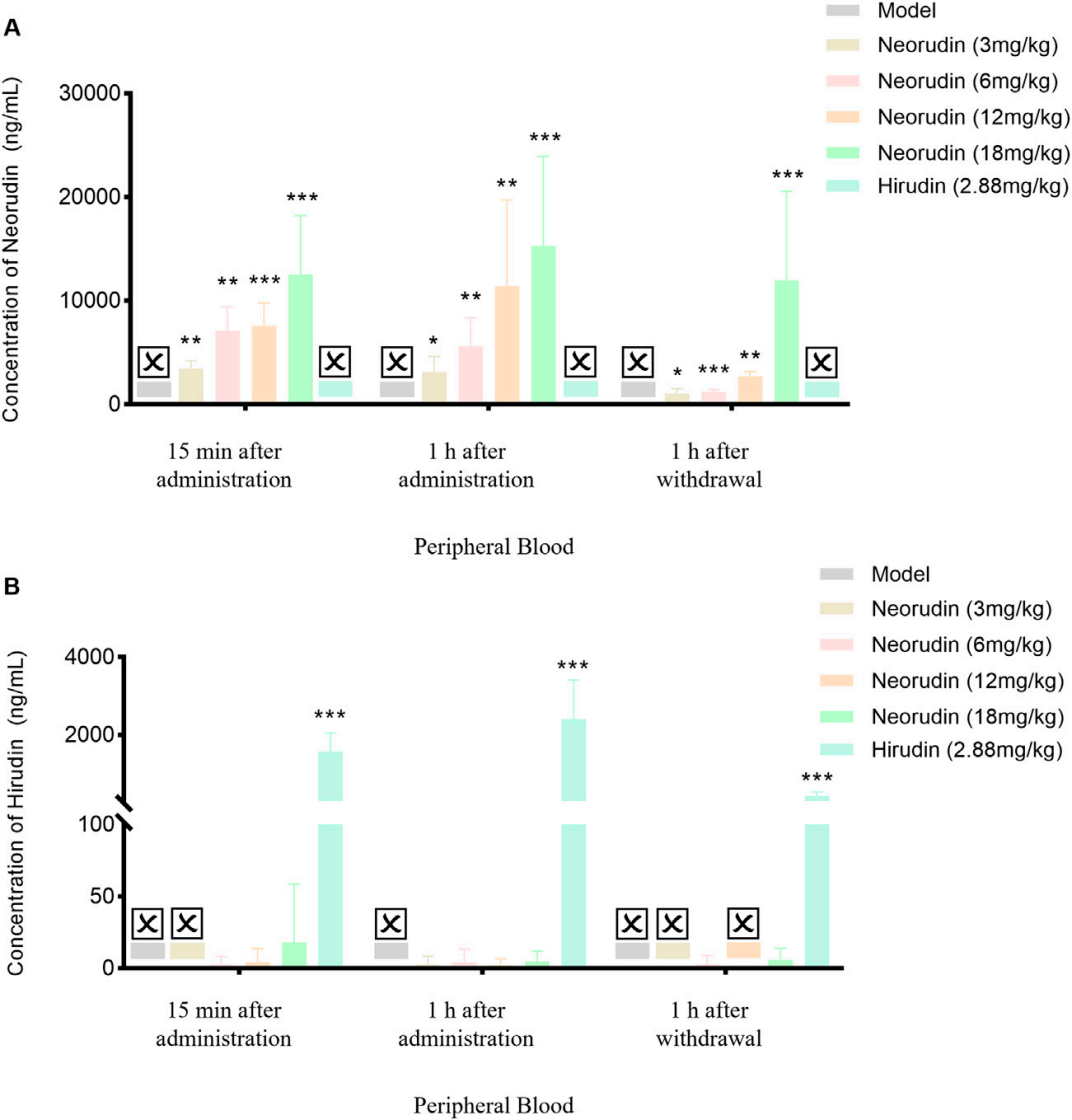
Changes of recombinant neorudin and hirudin in peripheral plasma (n = 5 rats per group). **(A)** Changes of neorudin in peripheral plasma. **(B)** Changes of hirudin content in peripheral plasma. (☒: None. Error bars, mean ± SEM from biological replicates. Compared to the model control group: *P < 0.05, **P < 0.01, ***P < 0.001).

The concentration of neorudin in the peripheral blood plasma of groups receiving 3 mg/kg, 6 mg/kg, 12 mg/kg, and 18 mg/kg showed an increasing trend with the dosage of neorudin administered during continuous administration for 15 min and 1 h, as well as at 1 h after discontinuation (Figure 3A). Specifically, its concentrations were 3,436 ng/mL, 7,106 ng/mL, 7,552 ng/mL, and 12,518 ng/mL for the 3 mg/kg, 6 mg/kg, 12 mg/kg, and 18 mg/kg groups after 15 min of administration, respectively. Then its concentrations decreased to 3,112 ng/mL, 5,592 ng/mL, 11,416 ng/mL, and 15,290 ng/mL after 1 h of administration. One hour after discontinuation, the concentration of neorudin in the peripheral blood plasma for the 3 mg/kg, 6 mg/kg, 12 mg/kg, and 18 mg/kg groups decreased significantly to 1,056.4 ng/mL, 1,216 ng/mL, 2,692 ng/mL and 11,950 ng/mL compared to the concentration after 1 h of administration.

During continuous administration for 15 min and 1 h, a small amount of hirudin was detectable in the peripheral blood plasma of the 3 mg/kg, 6 mg/kg, 12 mg/kg, and 18 mg/kg neorudin groups, with the concentration slightly increasing with the dose. Specifically, its concentrations were 0 ng/mL, 2.5 ng/mL, 4.3 ng/mL, and 18.1 ng/mL for the 3 mg/kg, 6 mg/kg, 12 mg/kg, and 18 mg/kg neorudin groups after 15 min of administration, respectively. However, a higher concentration of 1,570 ng/mL was detected in the hirudin group. The hirudin concentrations were 2.6 ng/mL, 4.1 ng/mL, 2.1 ng/mL, and 5 ng/mL for the respective neorudin groups after 1 h of administration, with a higher concentration of 2,400 ng/mL in the hirudin group. One hour after discontinuation, only the concentration of hirudin in the peripheral blood plasma of the 6 mg/kg and 18 mg/kg neorudin group was detected, with values of 2.8 ng/mL and 5.9 ng/mL, respectively. The hirudin group showed a higher concentration of 438 ng/mL (Figure 3B).

#### Detection of neorudin and hirudin in perithrombus plasma

**FIGURE 4:**
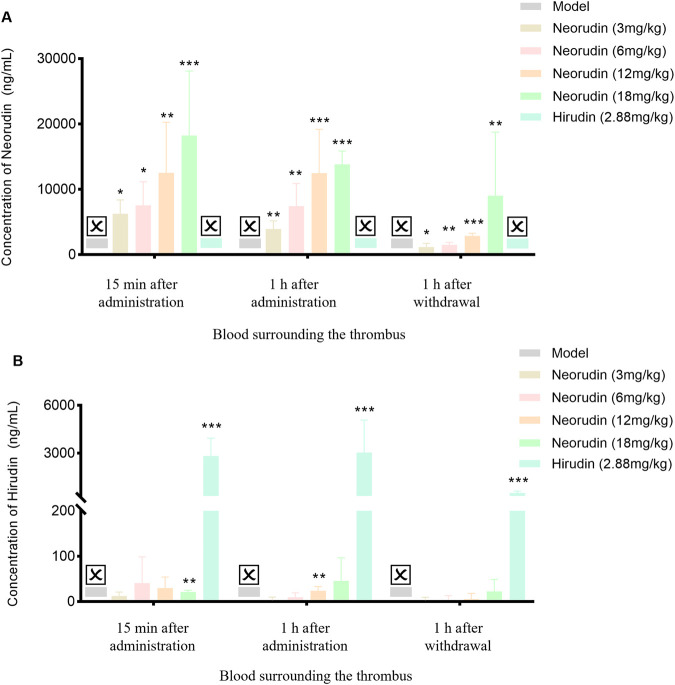
Changes of recombinant neorudin and hirudin in blood surrounding the thrombus (n = 5 rats per group). **(A)** Changes of neorudin in plasma surrounding the thrombus. **(B)** Changes of hirudin content in plasma surrounding the thrombus. (☒: None. Error bars, mean ± SEM from biological replicates. Compared to the model control group: *P < 0.05, **P < 0.01, ***P < 0.001).

Similar to the result of in peripheral blood plasma, the concentration of neorudin in the plasma surrounding the thrombus for doses of 3 mg/kg, 6 mg/kg, 12 mg/kg, and 18 mg/kg showed an increasing trend during continuous administration for 15 min and 1 h, and at 1 h after discontinuation (Figure 4A). Specifically, its concentrations were 6,270 ng/mL, 7,568 ng/mL, 12,528 ng/mL, and 18,226 ng/mL for the 3 mg/kg, 6 mg/kg, 12 mg/kg, and 18 mg/kg groups after 15 min of administration, respectively. Then its concentrations decreased to 3,926 ng/mL, 7,430 ng/mL, 12,492 ng/mL, and 13,840 ng/mL for the respective doses after 1 h of administration. One hour after discontinuation, the concentration of neorudin in the plasma surrounding the thrombus for the 3 mg/kg, 6 mg/kg, 12 mg/kg, and 18 mg/kg doses decreased significantly compared to the concentration after 1 h of administration, with values of 1,178.6 ng/mL, 1,521 ng/mL, 2,886 ng/mL and 9,038 ng/mL, respectively.

In contrast to neorudin, a small amount of hirudin was detectable in the plasma surrounding the thrombus for the 3 mg/kg, 6 mg/kg, 12 mg/kg, and 18 mg/kg neorudin groups, during continuous administration for 15 min and 1 h, and at 1 h after discontinuation, with the concentration slightly increasing with the dose. Specifically, during continuous administration for 15 min, its concentrations were 12.4 ng/mL, 40.8 ng/mL, 29.8 ng/mL, and 21.3 ng/mL for the respective doses. Then its concentrations were 3 ng/mL, 9.7 ng/mL, 23.8 ng/mL, and 45.5 ng/mL after 1 h of administration, respectively. One hour after discontinuation, the concentrations of hirudin in the plasma surrounding the thrombus for the 3 mg/kg, 6 mg/kg, 12 mg/kg, and 18 mg/kg doses decreased significantly compared to the concentration after 1 h of administration but was still detectable, with values of 3 ng/mL, 4.1 ng/mL, 5.6 ng/mL and 22.4 ng/mL, respectively. In comparison, a higher concentration of hirudin was detected in the 2.88 mg/kg hirudin group (Figure 4B). During continuous administration for 15 min, its concentration was 2,818 ng/mL and 3,042 ng/mL after 1 h of administration. One hour after discontinuation, its concentration was 502.6 ng/mL (Figure 4B).

#### Detection of neorudin and hirudin in thrombus homogenate

**FIGURE 5:**
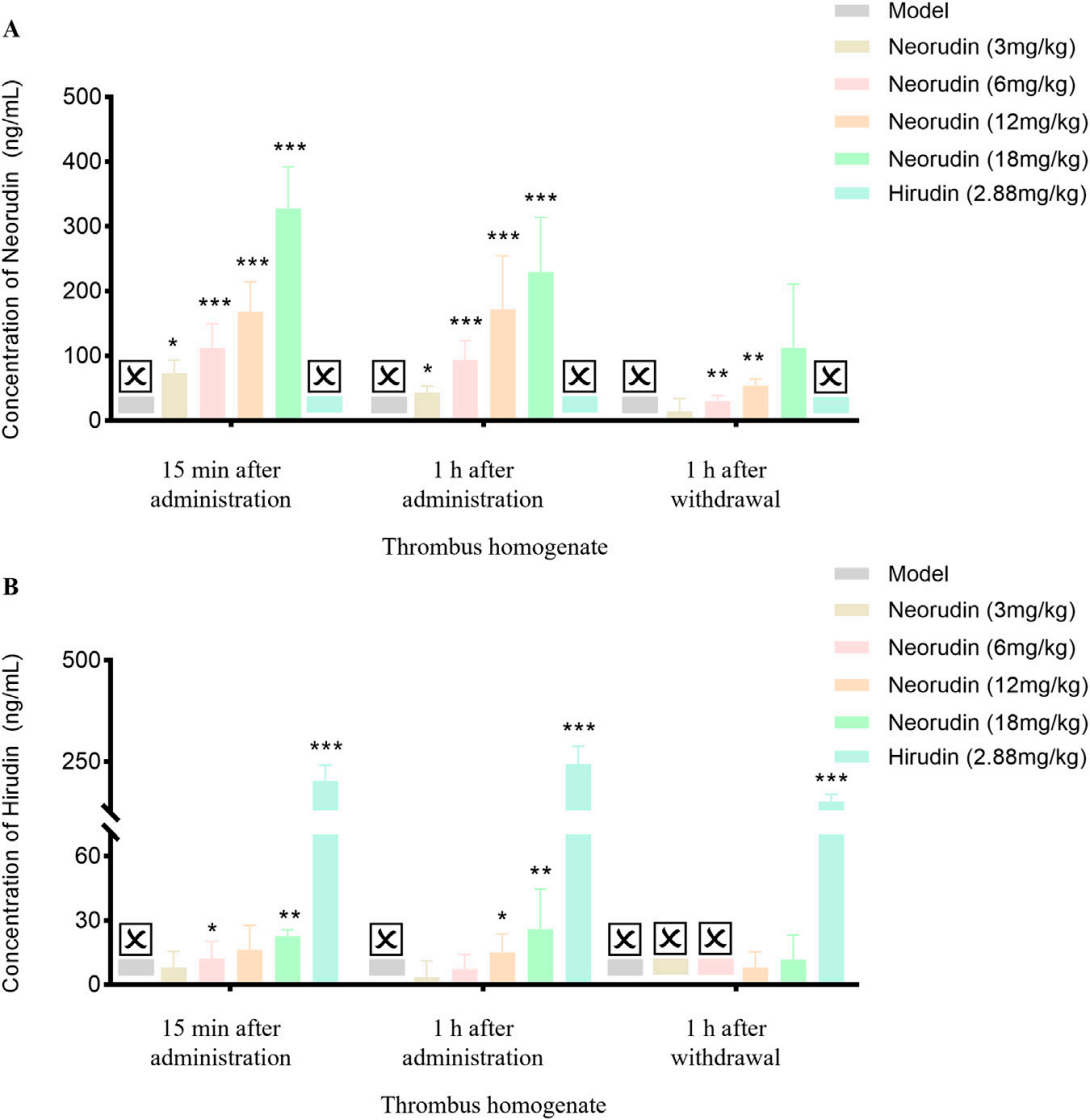
Content changes of recombinant neorudin and hirudin in thrombus homogenate (n = 5 rats per group). **(A)** Changes of neorudin in thrombus homogenate. **(B)** Changes of hirudin content in thrombus homogenate. (☒: None. Error bars, mean ± SEM from biological replicates. Compared to the model control group: *P < 0.05, **P < 0.01, ***P < 0.001).

In spite of a low value, the concentration of neorudin in the thrombus homogenate for doses of 3 mg/kg, 6 mg/kg, 12 mg/kg, and 18 mg/kg still showed an increasing trend during continuous administration for 15 min and 1 h, and at 1 h after discontinuation (Figure 5A). During a 15 min period of continuous administration, the concentrations of neorudin in the thrombus homogenate were 73.3 ng/mL, 112.9 ng/mL, 168.4 ng/mL, and 327.8 ng/mL for the respective doses. When the administration was extended to 1 h, its concentrations switched to 43 ng/mL, 93.9 ng/mL, 172.3 ng/mL, and 53.9 ng/mL, respectively. One hour after discontinuation, its concentrations were 14.4 ng/mL, 30.3 ng/mL, 53.9 ng/mL, and 112.5 ng/mL for the respective doses.

During continuous administration for 15 min and 1 h, a small amount of hirudin was detectable in the thrombus homogenate for the 3 mg/kg, 6 mg/kg, 12 mg/kg, and 18 mg/kg neorudin groups, with the concentration showing an increasing trend with the dose. Specifically, during the 15-min administration, its concentrations were 8 ng/mL, 12.2 ng/mL, 16.3 ng/mL, and 22.6 ng/mL for the respective doses. After 1 h of administration, its concentrations were 3.5 ng/mL, 7.3 ng/mL, 15.1 ng/mL, and 25.9 ng/mL, respectively. One hour after discontinuation, only the concentrations of hirudin in the thrombus homogenate for the 12 mg/kg and 18 mg/kg neorudin doses were detectable, with values of 8 ng/mL and 11.8 ng/mL, respectively. In contrast, a higher concentration of hirudin was detected in the 2.88 mg/kg hirudin group. During the 15-min administration, its concentration was 203.2 ng/mL, and increased to 244 ng/mL after 1 h of administration. One hour after discontinuation, its concentration decreased to 151.6 ng/mL (Figure 5B).

#### Distribution of neorudin and hirudin in different locations

**FIGURE 6:**
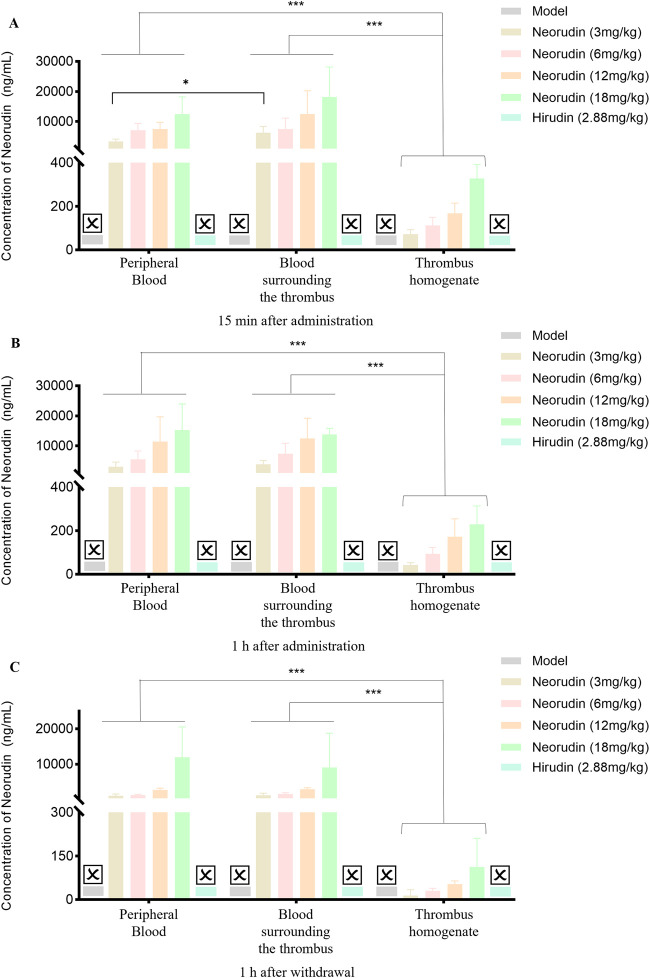
Distribution of recombinant neorudin in different locations within peripheral plasma, blood surrounding the thrombus, and thrombus homogenate at different time points after administration (n = 5 rats per group). **(A)** Distribution of neorudin at different locations 15 min after continuous administration. **(B)** Distribution of neorudin at different locations 1 h after continuous administration. **(C)** Distribution of neorudin at different locations 1 h after withdrawal of the drug. (☒: None. Error bars, mean ± SEM from biological replicates. *P < 0.05, ***P < 0.001).

At different time points, including administration for 15 min and 1 h, and at 1 h after discontinuation, the concentration of neorudin in each neorudin dosage group was similar between the peripheral plasma and the blood surrounding the thrombus, but was the lowest in the thrombus homogenate. During the 15-min administration, neorudin concentration in the blood surrounding the thrombus was notably higher compared to the peripheral plasma for all dosages. Specifically, in the 18 mg/kg group, its concentration was 18,226 ng/mL in the thrombus-surrounding blood versus 12,518 ng/mL in the peripheral plasma, with the lowest concentration in the thrombus homogenate at 327.8 ng/mL (Figure 6A). After 1 h of administration, the trend continued. In the 12 mg/kg group, neorudin was present at 12,492 ng/mL in the blood surrounding the thrombus, compared to 11,416 ng/mL in the peripheral plasma, and 172.3 ng/mL in the thrombus homogenate (Figure 6B). One hour post-discontinuation, its concentrations in the blood surrounding the thrombus remained still higher. The 12 mg/kg group showed 2,886 ng/mL in the thrombus-surrounding blood, compared to 2,692 ng/mL in the peripheral plasma, and 53.9 ng/mL in the thrombus homogenate (Figure 6C).

**FIGURE 7:**
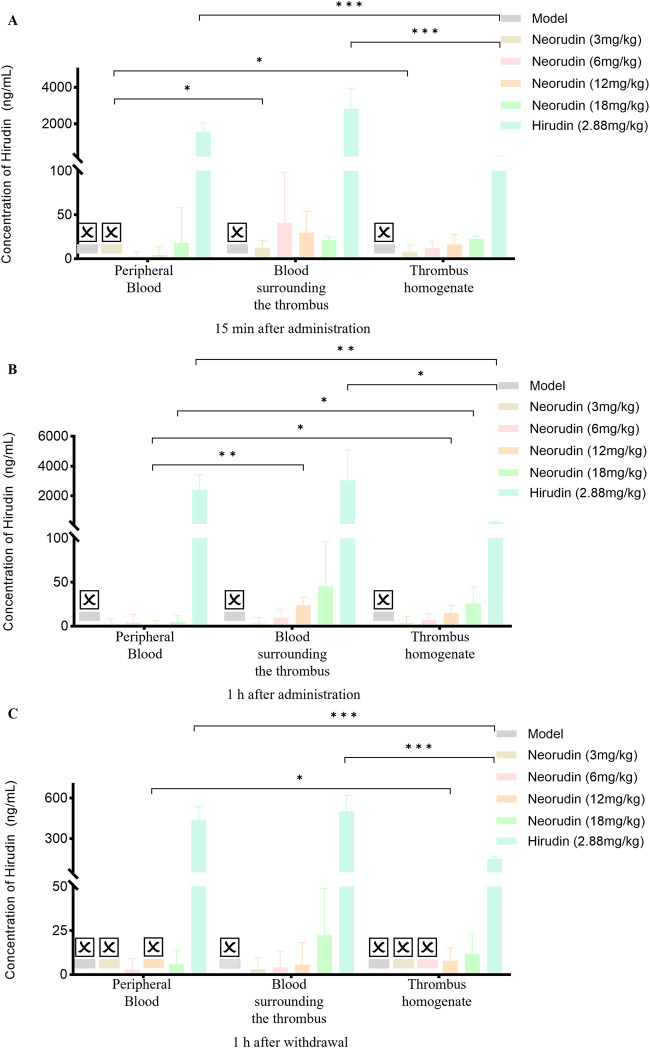
Distribution of hirudin in different locations within peripheral plasma, blood surrounding the thrombus, and thrombus homogenate at different time points after administration (n = 5 rats per group). **(A)** Distribution of hirudin at different locations 15 min after continuous administration. **(B)** Distribution of hirudin at different locations 1 h after continuous administration. **(C)** Distribution of hirudin at different locations 1 h after withdrawal of the drug. (☒: None. Error bars, mean ± SEM from biological replicates. *P < 0.05, **P < 0.01, ***P < 0.001).

Different from the distribution of neorudin, the concentration of hirudin for the 3 mg/kg neorudin group was significantly higher in the thrombus homogenate (8 ng/mL) and the blood surrounding the thrombus (12.4 ng/mL) during continuous administration for 15 min, compared with in the peripheral plasma (0 ng/mL) (Figure 7A). For the 12 mg/kg group during continuous administration for 1 h, the concentration of hirudin in the thrombus homogenate was 15.1 ng/mL and 23.8 ng/mL in the blood surrounding the thrombus, while 2.1 ng/mL in the peripheral plasma (Figure 7B). Moreover, the concentration of hirudin in the thrombus homogenate for the 18 mg/kg group was 25.9 ng/mL and 45.5 ng/mL in the blood surrounding the thrombus, higher than that in peripheral plasma (5 ng/mL) during continuous administration for 1 h (Figure 7B).For the 12 mg/kg group at 1 h after drug discontinuation, its concentration in the thrombus homogenate was 8 ng/mL and 5.6 ng/mL in the blood surrounding the thrombus, compared to 0 ng/mL in the peripheral plasma (Figure 7C). However, the concentration of hirudin in the hirudin group was the lowest in the thrombus homogenate during continuous administration for 15 min (203.2 ng/mL) and 1 h (244 ng/mL), and at 1 h after discontinuation (151.6 ng/mL), compared with peripheral plasma and the blood surrounding the thrombus. (Figure 7).

## Discussion

This study provided valuable insights into the distribution and pharmacological properties of recombinant neorudin and its active metabolite, hirudin, in the context of thrombus formation and peripheral blood circulation. The findings underscored the targeted antithrombotic effect of neorudin and the systemic anticoagulant action of hirudin, which were consistent with previous studies on the use of these agents in the management of thrombotic disorders.

Recombinant neorudin significantly increased TT in both plasma surrounding the thrombus and in peripheral blood, while also effectively reducing the wet weight of the thrombus. However, neorudin did not significantly affect APTT and PT in peripheral blood. This dose-dependent inhibitory effect, evidenced by the significant reduction in thrombus wet weight, aligns with the results of other preclinical thrombus models (Liu et al., 2022a; Liu et al., 2022b; Wang et al., 2013).

The concentration of neorudin in peripheral blood plasm, perithrombus plasma as well as thrombus homogenate at 1 h after discontinuation increased in a dose-dependent manner with administration and quickly decreased after cessation of treatment. This result was consistent with the phase I clinical studies of recombinant neorudin resulting from its relatively short half-life (approximately 2 h), indicating that neorudin has advantageous pharmacokinetic properties to ensure the effectiveness and safety of anticoagulant therapy (Liu et al., 2021 *)*.

Further studies on the local release of hirudin, the active product of recombinant neorudin, revealed that the concentration of hirudin was highest at the thrombus site, with higher concentrations in perithrombotic plasma compared to peripheral plasma, indicating that neorudin was cleaved to form hirudin. Moreover, the high level of cleavage at the thrombus site further suggested that neorudin had better targeting at the thrombus site, while reducing bleeding side effects. These findings supported the specific binding and inhibitory effects of recombinant neorudin at the site of thrombus formation.

The similar concentrations of recombinant neorudin in peripheral blood and perithrombotic plasma, along with its detectable presence in thrombotic tissue, indicate good thrombus targeting. The differential distribution of recombinant neorudin and hirudin in thrombi and peripheral blood suggested a unique mechanism of action. However, the lower concentration of recombinant neorudin and the higher concentration of its active product, hirudin, in thrombus homogenate might be related to subsequent cleavage by FXa or FXIa and the “escape” after the binding of thrombin with neorudin/hirudin (Dong et al., 2018; Zhang et al., 2008).

The higher levels of hirudin, the active product of recombinant neorudin, in perithrombotic plasma and thrombotic homogenate compared to peripheral plasma further supported the local release of hirudin. These findings demonstrated good targeting, specific binding of hirudin to thrombin, its inhibitory effect on thrombus formation, and a reduced risk of bleeding side effects. These findings supported the specific binding and inhibitory effects of recombinant neorudin at the site of thrombus formation (Bichler et al., 1993).

However, the low levels of hirudin detected in the thrombus homogenate raised important questions about the binding dynamics between hirudin and clotting enzymes. It had been suggested that the majority of hirudin bound to thrombin in a molar ratio (Chang, 1990; Konno et al., 1988; Wakui et al., 2019), which resulted in a lower concentration of free hirudin detectable by current mass spectrometry methods. This limitation highlighted the need for advanced analytical techniques that could accurately quantify the levels of hirudin-thrombin complexes, as well as free hirudin, to gain a more comprehensive understanding of the pharmacological properties of hirudin. (Betz et al., 1991; Sohn et al., 2001).

Targeted anti-thrombotic hirudin had a minor impact on the coagulation system in non-thrombotic regions, reflecting the characteristic of traditional Chinese medicine (TCM) theory that “only the area with the ailment is affected.” Compared to other anticoagulants, it had significant advantages. This specificity not only reduced the risk of adverse effects but also provided a more effective method of thrombus management. Understanding the mechanism of action of hirudin in the thrombus formation process and its binding with thrombin was crucial for optimizing treatment strategies and developing novel anticoagulant drugs. (Chen et al., 2023; Maxwell et al., 2023; Stone and Payne, 2015; Thompson et al., 2017).

**FIGURE 8:**
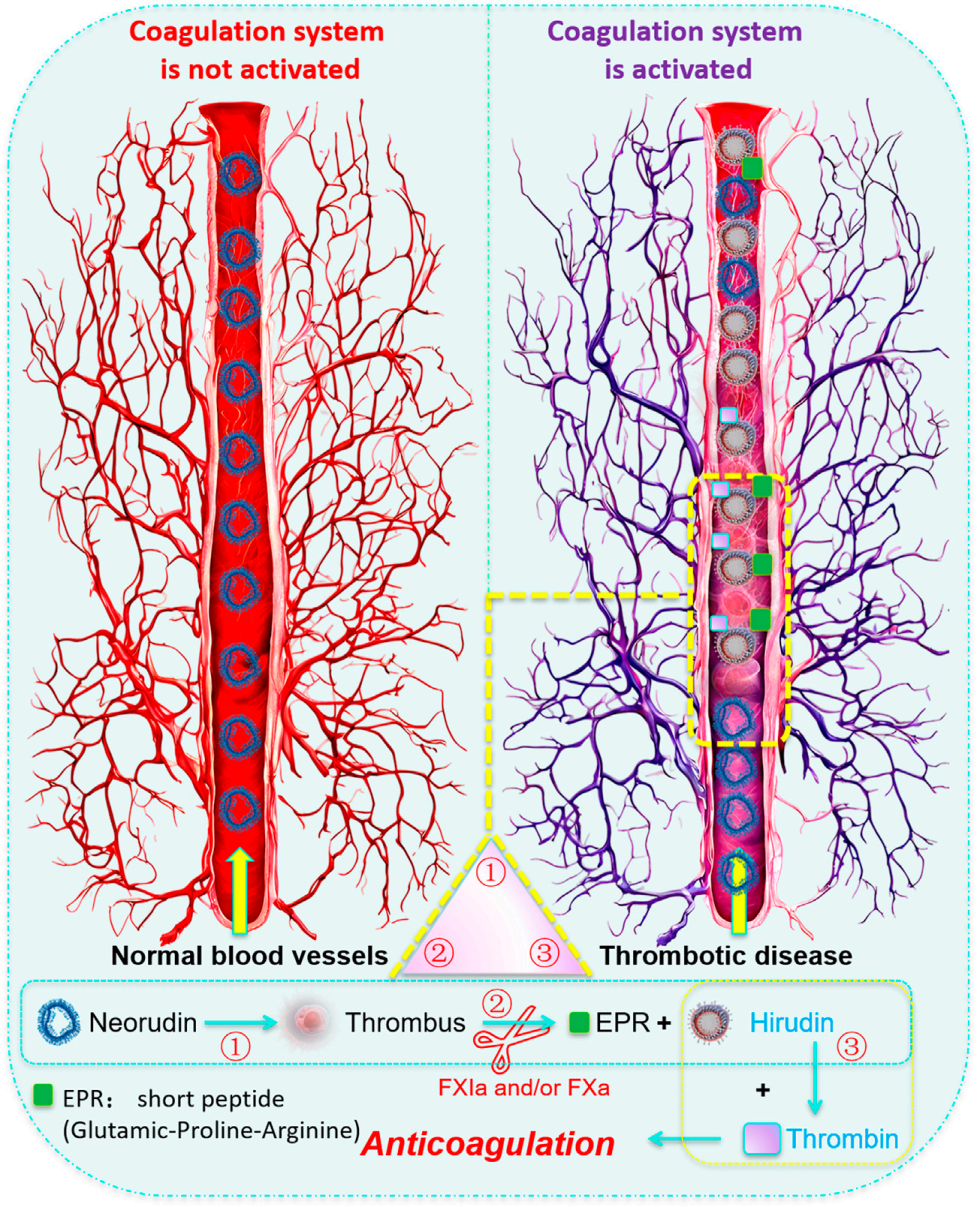
Cover Art: Schematic diagram of the local action mechanism of recombinant neorudin on thrombus. When local thrombosis formed *in vivo* and the coagulation system was activated, the activated coagulation factors FXa and/or FXIa could specifically recognize and enzymatically cleave neorudin, cutting off the N-terminal EPR short peptide from neorudin, transforming neorudin from an inactive form into an active anticoagulant hirudin. Hirudin then specifically bound to thrombin in a 1:1 ratio, achieving targeted antithrombotic action. That is, the complete neorudin lacks antithrombin activity, and only when the coagulation system in the body is activated can hirudin be released to produce antithrombotic activity.

In conclusion, this study significantly enhanced our understanding of the distribution and pharmacological properties of recombinant neorudin and its active metabolite hirudin within the contexts of thrombus formation and peripheral blood circulation (Figure 8). It underscored the targeted antithrombotic effect of recombinant neorudin and the systemic anticoagulant action of hirudin, aligning with and extending upon the findings of previous preclinical and clinical research. The study highlighted the necessity for further investigations aimed at developing advanced analytical methods capable of precisely quantifying both hirudin-thrombin complexes and free hirudin. Such research was crucial for a more comprehensive understanding of hirudin’s pharmacological properties and its interaction with thrombin, which will not only provided new insights and guidance for the treatment of thrombotic diseases but also supported the development of more effective anticoagulant therapies. This work, therefore, not only contributed to our knowledge base regarding the anticoagulant and antithrombotic mechanisms of recombinant neorudin and hirudin but also set a stage for future studies that aimed to optimize the management of thrombotic conditions.

## Data Availability

The raw data supporting the conclusions of this article will be made available by the authors, without undue reservation.

## Supplementary material

SUPPLEMENTARY FIGURE S1 Typical LC-MS/MS Chromatogram. A Spectrum of Neorudin. B Spectrum of Hirudin.
