# Supplementary figures and images for: Recombinant neorudin and its active metabolite hirudin: the fate in vivo of a novel anticoagulant drug
Source: Front Pharmacol. 2024 Sep 17;15:1443475. doi: 10.3389/fphar.2024.1443475 (PMC11442382; doi:10.3389/fphar.2024.1443475)

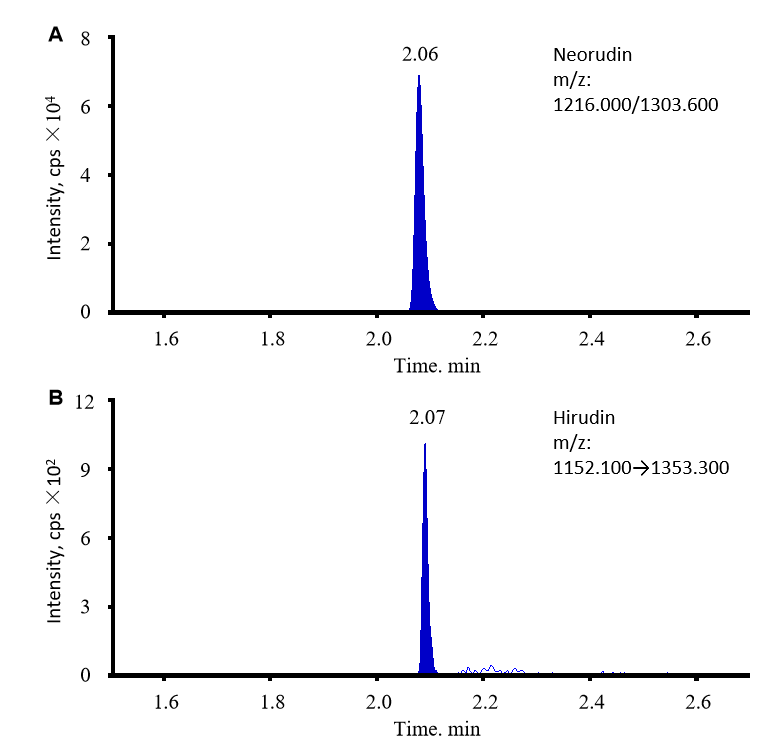

Supplement: Supplementary file 1 [file Image1.TIF]
